# Impact of an Interoception-Based Program on Emotion Regulation in Autistic Children

**DOI:** 10.1155/2022/9328967

**Published:** 2022-04-20

**Authors:** Kelly Mahler, Kerri Hample, Claudia Jones, Joseph Sensenig, Phoebe Thomasco, Claudia Hilton

**Affiliations:** ^1^Elizabethtown College, Elizabethtown, Pennsylvania, USA; ^2^Mahler Occupational Therapy, Elizabethtown, Pennsylvania, USA; ^3^University of Texas Medical Branch, Galveston, Texas, USA

## Abstract

**Purpose:**

The aim of this study was to determine the feasibility and effectiveness of a 25-week school-based intervention and its ability to improve interoception and emotion regulation in an autistic pediatric population.

**Method:**

One-group pre- and posttest design implementing *The Interoception Curriculum: A Guide to Developing Mindful Self-Regulation* in a self-contained school. Participants were 14 (11 male, 3 female) students between 9 and 19 years old. The Behavior Rating Inventory of Executive Function 2 (BRIEF-2) and the Caregiver Questionnaire for Interoceptive Awareness-2nd Edition (CQIA-2) were used to determine changes in interoceptive awareness and emotion regulation.

**Results:**

Statistically significant improvements were found between the preintervention and postintervention scores for both interoceptive awareness and emotion regulation.

**Conclusion:**

This was the first study to examine the Interoception Curriculum in its entirety, providing evidence that the use of the Interoception Curriculum is feasible in a school setting and suggests that this intervention is effective for improvement of interoception. Findings also suggest that this improvement in interoception is related to improvement in emotional regulation for an autistic pediatric population.

## 1. Introduction

### 1.1. Impact of an Interoception-Based Program on Emotion Regulation in Autistic Children

Emerging literature suggests interoception is a sensory system that is commonly impacted in autistic individuals, ultimately affecting emotional regulation, social participation, and occupational performance [1–4]. Interoception allows one to perceive the physiological state of their body, such as recognizing signals of respiratory effort, temperature, fatigue, hunger, thirst, satiety, pain, muscle aches, and heart rate [5, 6]. When interoceptive cues go unnoticed or are misunderstood, it can lead to a construct known as alexithymia [7, 8], or the reduced ability to identify or describe emotions. Alexithymia often coexists with autism spectrum disorder [9]. As characteristics of autism spectrum disorder (ASD) already complicate one's ability to participate in daily activities, alexithymia may further reduce an autistic individual's ability to understand, verbalize, and respond to their emotions due to underlying interoceptive confusion [1]. Ultimately, this impacts the ability to successfully regulate emotions [4, 7].

A small but growing body of research has begun investigating the use of an interoception-based approach to improve emotional regulation [2]. This study examined the emotional regulation outcomes in a pediatric autistic population using an interoception-based approach.

## 2. Literature Review

According to the DSM-5, criteria necessary for a diagnosis of ASD include impaired social interaction and communication and repetitive behaviors often associated with ASD [10]. Although not included in the diagnostic criteria, alexithymia, or difficulty identifying and understanding emotions in self, is particularly relevant in the autistic population, impacting up to 85 percent of individuals [11]. Furthermore, also not included in the DSM-5 criteria, but perhaps related to the alexithymia experience, are differences in emotional regulation. A significant challenge often reported by autistics, the ability to regulate emotions, includes identifying and understanding one's emotions as well as using strategies to increase or decrease responses when necessary [2, 12, 13].

In the school setting, disruption in emotional understanding can affect a student's success in regulating classroom behaviors, such as their ability to attend to classroom activities, participate in group assignments, solve problems, complete schoolwork, and feel safe in their learning environments [2, 13, 14]. It is likely that disruption in emotional regulation is also a key contributor to the outward behaviors in autistic children, such as aggression, meltdowns, shutdowns, and self-injury [13]. Because negative school experiences are pervasive for many autistic students [15], successful support strategies are necessary.

### 2.1. Interoception and Emotional Awareness

Because autistic individuals often display outward behaviors indicative of emotional regulation challenges, research has begun to examine underlying mechanisms that may contribute to these behaviors. One of the mechanisms under investigation is interoception [16]. Findings from research have identified the insular cortex, or the insula, as the brain's main integration center for interoception, in which sensory messages are translated into a corresponding homeostatic emotion, such as detecting body temperature, pain, hunger, fullness, sexual arousal, and identification of affective states such as excitement, frustration, and anxiety [5, 17, 18]. Interoceptive awareness has been shown to be related to emotion regulation [19]. It is necessary for identifying internal physiological processes related to affective feeling and is a means of integrating bodily sensations, cognitive processes, and emotional feeling. The insula's vast connections enable it to provide conscious awareness of emotions and the corresponding underlying bodily changes [20]. Atypical functional activation, as well as connectivity of the insula in ASD, is widely documented [21]. Having access to interoceptive information allows an individual to be aware of an emotion cue early and to process, interpret, and strategize at the onset of stressful events before the stress response intensifies [22].

### 2.2. Interoception, Alexithymia, and Emotion Regulation

Effective interoceptive processing drives clear awareness of homeostatic and affective emotions, and this emotional awareness has been found to underlie successful emotional regulation [23]. When one is unable to notice or understand their interoceptive body signals, crucial clues are missed that allow for accurate identification of emotions. This interoceptive confusion may contribute to alexithymia, a construct that presents with difficulty in identifying and describing affective emotions [7, 8]. Frequently, alexithymia coexists with clinical disorders associated with poor interoceptive awareness and emotional regulation such as anxiety disorders, mood disorders, and eating disorders [24–26]. Interoception confusion has also been identified in autistic individuals with alexithymia [6–8, 27].

Because emotional awareness is critical to emotion regulation, individuals who are not able to delineate specific emotions are often unsuccessful in effectively regulating their emotional responses. For example, identifying a feeling such as fear, hunger, or fatigue will provide the information needed to know what the body needs for comfort and regulation (e.g., seeking safety when fearful, eating when hungry, or taking a nap when fatigued). Therefore, addressing the underlying interoceptive challenges preventing successful identification and interpretation of emotions is needed, which could result in enhanced success in emotion regulation and behaviors [2, 7].

### 2.3. Current Study

The aim of this research is to determine the effectiveness of a 25-week interoception-based intervention for improving emotion regulation in autistic children. This is a follow-up to a previous 8-week occupational therapy intervention study based on *The Interoception Curriculum: A Guide to Developing Mindful Self-Regulation* (IC) [2, 28]. Results indicated statistically significant improvements in the participants' emotion regulation abilities.

Because the pilot intervention lasted eight weeks, only a portion of the IC was completed [2]. Given 25 weeks in the current study, the full curriculum could be completed. Due to the statistically positive impact of the previously completed eight-week version, we hypothesize that the retrospective data analysis from the 25-week study will also indicate improvement in emotion regulation and interoceptive awareness. The aim of this study was to examine feasibility and determine the effectiveness of a 25-week intervention based on *The Interoception Curriculum: A Guide to Developing Mindful Self-Regulation* (IC) in improving interoception and emotion regulation in a pediatric autistic population. Identity-first language (e.g., autistic individual) will be adhered to throughout this paper because autistic individuals, advocates, and scholars assert that person-first language (e.g., individual with autism) reflects an ableist perspective and contributes to disability stigma [29, 30].

## 3. Methodology

In this study, we conducted a retrospective analysis that utilized a 25-week one-group pre- and posttest design implementing *The Interoception Curriculum: A Guide to Developing Mindful Self-Regulation* (IC) [28] in a self-contained school for autistic children that included children who were temporarily enrolled because of difficulties in their home schools. Ethical approval for this study was obtained from the Institutional Review Board at Elizabethtown College (#1143287-2). Appropriate consents and assents were obtained from all participants and parents.

### 3.1. Participants

Two recruitment standards were utilized: one for the therapists who would be involved in the study to implement the protocol and another for the children who were recruited to engage in the interoception intervention.

#### 3.1.1. Occupational Therapists

Pediatric occupational therapists with a prior knowledge of interoception were recruited through convenience sampling using a uniform post on a social media platform. The inclusion criteria for occupational therapists (OTs) were as follows: (1) currently work with children within a school-based setting, (2) work with a caseload of children who fall within the student criteria, (3) able to complete the intervention during the study timeframe, and (4) employed at a site which afforded consent for the inquiry. The first professional to respond who met the following inclusion criteria was a social worker, however, an occupational therapist, who met the above inclusion criteria was involved throughout the entire process.

#### 3.1.2. Autistic Children

The social worker and OT then used network sampling to recruit children from their caseloads who met the following criteria: (1) parents who were willing to sign the consent form, (2) able to provide assent, (3) learning in a facility that provided site consent, (4) had completed pre- and post-test assessments, (5) were on the OT caseload, and (6) who were identified with perceived delays in sensory processing and/or interoceptive awareness as determined by therapist discretion. Children were excluded from this study if they did not meet the above inclusion criteria. The intervention occurred with the pediatric participants via their service professionals who obtained the staff training (described in Intervention).

### 3.2. Assessments

Two outcome measures were selected to examine interoceptive awareness and emotion regulation: the Behavior Rating Inventory of Executive Function, 2^nd^ edition® (BRIEF-2®; [31]) and the Caregiver Questionnaire of Interoceptive Awareness subtest of the Comprehensive Assessment of Interoceptive Awareness-2^nd^ edition (CQIA-2; [32]).

#### 3.2.1. Behavior Rating Inventory of Executive Function, 2^nd^ edition (BRIEF-2; [31])

Emotional, behavior, and cognitive regulation data were collected using the BRIEF-2, a standardized valid and reliable questionnaire that includes subcategories of shift and emotional control. This evaluation assesses everyday behaviors associated with emotional, behavior, and cognitive regulation in the home and school environments. For example, the category of *shift* addresses the ability to transition from one activity to another and *emotional control* focuses on the ability to handle changes in emotions. Higher scores indicate greater executive function impairment. Because this study was conducted in the school context and emotion regulation at school was the targeted behavior, the Teacher Form of the BRIEF-2 was utilized. The BRIEF-2 has test-retest reliability for the indexes and composite score all above 0.80 (BRI = 0.83, ERI = 0.88, CRI = 0.89, and GEC = 0.90) within a two- to three-week time span, indicating that the BRIEF-2 was an acceptable measurement tool for test-retest purposes [31]. This test has also been found to be reliable and valid in specific populations including children with attention deficit hyperactivity disorder (ADHD), anxiety, learning disabilities, and ASD making it suitable for the participants in this study [31].

#### 3.2.2. Caregiver Questionnaire for Interoceptive Awareness, 2^nd^ edition (CQIA-2; [32])

School staff reported their perceptions of the student's interoceptive awareness using the CQIA-2. The CQIA-2 consisted of twenty-four Likert-scale questions on which caregivers reported the frequency in which they observed a certain interoception-related behaviors (e.g., can the student figure out exactly how they feel?; is the student able to tell when they need to take a break before feelings become too much?; does the student recognize when they need to go to the bathroom in a timely manner in order to avoid accidents or last-minute emergencies?; and does the student seek out or request food on their own when hungry rather than needing to be reminded to eat?). After analysis of questions included in the CQIA-2, two broad categories of questions, *homeostatic* and *general* were created based on the questions' contents. Included in the *homeostatic* category were responses related to *sleep*, *eating*, *toileting*, *pain and healthcare management*, and *thirst*. The *general* category represented questions broader in nature (not mentioning specific emotion words), which was further divided into *emotional response* and *emotional awareness*.

Raw scores from the Likert-scale questions of the CQIA-2 were combined to receive an overall score for each student. Further, the raw scores were totaled for each category of *homeostatic* and *general*, as well as for the subcategories, consisting of *sleep*, *toileting*, *eating*, *pain and healthcare management*, *thirst*, *emotional response*, and *emotional awareness*. Higher scores were indicative of greater perceived interoception awareness. While this test is relatively new and rigorous psychometric analysis is needed, there are no other interoception-based caregiver reports currently available.

#### 3.2.3. Assessment Completion

To maintain consistency, for each study participant, the school staff member who completed the pretest CQIA-2 and BRIEF-2 forms was also required to complete the posttest forms. Researchers received all pre- and postassessments through a secure mailing address. Complete assessments were stored in a locked file cabinet in a locked office of principal investigator before and after being analyzed for the results of the study. Researchers communicated missing information to the primary occupational therapist, and if they were unable to provide complete assessments, the participant was excluded from the study. Participant confidentiality was maintained by omitting the names from all assessment forms. All electronic data were password protected and stored by only the principal investigator.

#### 3.2.4. Scoring Methods

Each assessment tool was scored by two of the researchers; first independently and followed by collaboration in pairs to compare scores to ensure interrater reliability and triangulation [33]. Once scores were confirmed, the data were stored on an excel sheet to later utilize during data analysis in a password protected computer. Uniform standards and directions were followed when scoring.

### 3.3. Staff Training

The involvement of the primary occupational therapist was required throughout the assessment and intervention phases, serving at minimum as a weekly consultant or support to the personnel implementing the assessment/intervention (e.g., classroom staff). All staff involved in the implementation of the study were required to participate in an initial, virtual four-hour interoception training that was provided by study researchers. Training was mandatory for the primary occupational therapist and all personnel who were involved in the assessment and/or intervention process. The initial training provided education regarding interoception, an overview of the study procedures, and details needed to perform pretest assessments uniformly and accurately.

To further ensure fidelity of implementation, the staff members were each provided with a copy of the IC instructional manual and access to premade visual and language supports that they were able to use in their original form or slightly adapted (e.g., reducing the number of interoception descriptor words presented on one page) to ensure a best match for each study participant. Throughout the assessment and intervention phases, additional support and communication were provided to the school staff via email, phone, or a closed virtual platform. The feedback and questions posed by the school staff suggested a high degree of understanding of the program and their motivation level of implementation appeared high throughout the intervention phase. To the best of our knowledge, the intervention, as described below, was implemented according to the original plan presented during the initial staff training but a fidelity measure was not included.

### 3.4. Intervention

The IC included 25 sequential lesson plans that were divided into three sections: *body lessons*, *emotion lessons*, and *action lessons [*28*]*. The *body lessons* were designed to teach the individual to notice body signals in a variety of body parts using body mindfulness strategies that were carefully adapted to meet a variety of cognitive, attentional, and learning abilities. The *emotion lessons* were designed to help each participant use the body signals noticed as clues to their unique emotional experience. The *action lessons* were designed to help each participant explore and discover feel-good actions that promoted comfort within each identified body-emotion connection. Each lesson was implemented during each school day by school staff across one week for a total weekly time of approximately 30-60 minutes per lesson.

Consistent with other studies shown to enhance interoceptive understanding, many of the strategies included in the IC are associated with the practice of body mindfulness (e.g., [34]). To make it more accessible to a wider variety of learners, the strategies in the IC are adapted forms of body mindfulness. For example, participants are invited to notice sensations in a single body part while playfully engaging in an activity that evokes a stronger sensation thus helping to capture their attention and interest (e.g., noticing how the hands feel while playing in water and squeezing putty). Also, visual and language supports were provided throughout the activities to help support communication and develop interoception vocabulary. Although the traditional methods of body mindfulness have been adapted, the goal of the IC supports the same outcome: cultivating inner curiosity, awareness, and understanding for the purpose of enhanced emotion regulation.

### 3.5. Data Analysis

After verification of the participants' scores for the BRIEF-2 and CQIA-2, the data were analyzed using SPSS Statistics (Version 26; IBM Corporation, Armonk, NY). The Wilcoxon signed-rank test was used to complete the analyses due to lack of normality in the data and the study's small sample size [35]. After the analyses were completed, BRIEF-2 scores and CQIA-2 scores for each participant were critiqued by each researcher to search for trends and similarities within the data. Participants with incomplete BRIEF-2 forms were excluded from the study. Effect sizes were examined to quantify the magnitude of the changes [36]. A Transparent Reporting of Evaluations with Nonrandomized Designs (TREND) statement checklist was used to assure adherence to transparent reporting of this study (see Supplementary TREND file (available here).)

## 4. Results

Participants ranged from the ages of nine to 19 years (*m* = 13.86, *SD* = 3.325). Twenty participants were enrolled in the study; and 14 datasets with complete prepost BRIEF-2 and CQIA-2 scores were obtained. Two BRIEF-2 forms had not been submitted by the teachers, and four of the participants returned to their home schools before the intervention was completed. All participants were white and had clinical diagnoses of ASD. Additionally, secondary educational diagnoses included speech-language impairment (*n* = 4), emotional behavioral disability (*n* = 2), intellectual disability (*n* = 3), and other health impairment (*n* = 1) (Table 1).

All BRIEF index *t* score changes were significant, and all subcategories yielded statistically significant changes between prestudy and poststudy *t* scores, except for *initiate* and *task-monitor* in the *cognitive index* group. Effect sizes were all categorized as very large except for shift (large), initiate (large), and task monitor (medium; [36]). Statistically significant improvements were seen in CQIA-2 scores for the *overall* score and broad *homeostatic* and *general* categories. Effect sizes were all very large except for eating (large), thirst (large), toileting (medium), and emotional awareness (medium) (see Table 2 for details). Changes in all subcategories were significant except for toileting and sleep (see Table 2 for details).

## 5. Discussion

This was the first study to examine the outcome after completion of the entire IC curriculum which was embedded directly into the school environment. It is aimed at determining the effectiveness of an interoception-based intervention on emotion regulation for autistic children. The results of this study indicate that use of the IC is feasible and was successful in improving emotion regulation for autistic children. This suggests that interoception confusion may be related to emotion regulation difficulties present in many autistic children. This interoception approach, which teaches how to notice and interpret body signals, may be an effective intervention for improving emotion regulation as well as the associated outward behaviors.

The participants in this study were reported to experience gains in both affective emotion regulation (e.g., results on BRIEF-2) and homeostatic emotion regulation (e.g., per improvement in eating, thirst, and pain on CQIA-2). While affective emotion regulation is widely studied and supported in existing autism approaches (e.g., regulation of anxiety or frustration), focus on homeostatic emotion regulation is limited. After completing the IC, which takes a more holistic approach to supporting interoceptive growth for the purposes of understanding and regulating both affective and homeostatic emotions, participants showed significant improvements in areas related to both affective and homeostatic emotion regulation. These findings suggest that a more global exploration of the emotional experience should be considered and further studied in emotion regulation intervention. The cognitive changes elicited by the intervention appear to have enhanced the connections of the insula of the brain to provide conscious awareness of emotions and the corresponding underlying bodily changes supporting more socially acceptable emotion expression choices by the study participants [22], according to teacher reports.

The significant improvements found in emotion regulation, emotion awareness, and emotion response in this study suggest the possibility that interoceptive confusion may contribute to alexithymia, a construct that presents with difficulty in identifying and describing affective emotions [7, 8]. Additionally, the findings provide some preliminary support that an interoceptive approach, such as the IC, may improve the alexithymia experience. Because alexithymia frequently coexists with clinical disorders outside of autism, studying the use of the IC in other populations such as individuals with anxiety disorders, mood disorders, and eating disorders may be promising. Directly measuring alexithymia in these future studies may prove helpful, especially in populations that can self-report.

### 5.1. Limitations

Though this research shows promise for interoception-based interventions for autistic children, this study had several limitations. First, the outcome measures used presented limitations because both the BRIEF-2 and the CQIA-2 are caregiver reports which are subjective and may lead to bias in the answers provided. In addition, the answers are from the school staff perspective and not from the child's perspective. However, because the participants in our study did not have the communication skills needed for self-report via existing interoception interviews or questionnaires, we concluded that caregiver report was the most appropriate measure for our population. In addition, the 25-week school-based intervention, carried out in one school only, and all measures being directed by one occupational therapist, might bias the results of this study. However, having a multidisciplinary team involved in the intervention helped to reduce potential bias of the single occupational therapist directing the program. Also, the explicit, direct attention, and targeted support for these autistic children might have accounted for the improvement. With no follow-up data, it is not possible to evaluate how robust these changes are sustained over time.

In addition to bias, when referencing the CQIA-2, it should be noted that this outcome measure is relatively new; therefore, the construct validity is not fully known. However, the BRIEF-2 is a well-respected, standardized, interdisciplinary tool that specifically examines emotional regulation, therefore suggesting that the significant gains in emotional regulation are valid [37].

Regarding methodology, we had limitations related to control, time, sample size, and generalizability. This study was not a randomized control trial and did not include a control group [38], and the sample size was relatively small.

### 5.2. Recommendations

Continued research in this area is warranted. Autistic individuals have high rates of emotional dysregulation, and the underlying causes are poorly understood. Based on the results of this study, interoception may be a contributing factor that deserves more attention in both research and clinical applications.

The study participants were autistic students with complex needs, enrolled in a self-contained school. Many of the participants were enrolled in this school because of high levels of dysregulation that could not be successfully supported by their home school districts. A program, such as the IC, which may potentially address the underlying emotion regulation needs in a more effective manner, could help students successfully participate in their most natural contexts.

The significant findings with the participant population of this study show promise for use of the IC with a variety of individuals with emotion regulation challenges. For individuals who are not reaching their full potential with their current treatment, interoception-based interventions could be an alternative option. Because of the short, 25-lesson duration and potential for inclusion as part of a school-based social-emotional program, this could eventually become a widely used, effective option. Future recommendations include randomized control trials or longitudinal studies, follow-up measures to examine sustainability of results, implementation of the IC in diverse contexts, and inclusion of participants of varying ages and diagnoses.

## 6. Conclusion

Results of this study suggest that use of the 25-week IC is feasible and suggest that an interoception-based approach can improve emotion regulation in autistic children. They also suggest that interoception is an important foundational component contributing to successful homeostatic and affective emotion regulation.

## Figures and Tables

**Table 1:** Demographics of participants.

*N*	14
Age (mean)	13.86 years
Age (SD)	3.325 years
Age (range)	9-19 years
Sex	11 male, 3 female

**Table 2:** Teacher report pre- and postintervention scores (*N* = 14).

BRIEF-2	Mean pre-*t* score (SD)	Mean post-*t* score (SD)	*Z* score	*p*	Effect size (*d*)
^∗∗^Behavior regulation index	79.87 (6.36)	73.40 (7.88)	-3.008	0.003	-2.704
^∗∗^Inhibit	77.73 (9.62)	71.20 (8.15)	-2.731	0.006	-2.136
^∗∗^Self-monitor	80.33 (6.03)	74.53 (9.00)	-2.767	0.006	-2.197
^∗∗^Emotional regulation index	87.67 (4.91)	80.07 (4.71)	-3.184	0.001	-1.836
^∗∗^Emotional control	85.53 (5.85)	76.46 (4.41)	-3.268	0.001	2.131
^∗∗^Shift	86.27 (6.01)	81.27 (5.64)	-2.852	0.004	-1.059
^∗∗^Cognitive regulation index	74.40 (8.82)	69.07 (6.34)	-2.767	0.006	-2.197
Initiate	68.33 (11.99)	63.80 (8.06)	-1.754	ns	-1.061
^∗∗^Working memory	73.00 (10.18)	68.67 (8.74)	-2.627	0.009	-1.972
^∗^Plan/organize	73.60 (8.55)	68.67 (6.09)	-2.135	0.033	-1.390
Task-monitor	70.27 (8.42)	67.80 (7.88)	-1.378	ns	-0.792
--^∗∗^Organization of materials	74.47 (8.40)	67.80 (9.25)	-3.239	0.001	-3.458
^∗∗^Global executive composite	81.53 (7.27)	74.07 (5.40)	-3.411	0.001	-4.436
CQIA-2					
^∗∗^Homeostatic	39.0 (7.42)	52.1 (5.93)	3.301	0.001	1.950
Sleep	4.9 (1.64)	6.0 (1.41)	1.798	ns	0.719
^∗^Eating	10.7 (3.13)	13.1 (2.45)	2.842	0.04	0.854
Toileting	9.1 (1.83)	9.9 (.52)	1.867	ns	0.595
^∗∗^Pain/healthcare management	8.1 (3.24)	14.0 (2.88)	3.190	0.001	1.925
^∗∗^Thirst	6.3 (3.37)	8.9 (1.67)	2.821	0.003	0.978
^∗^General	13.9 (2.57)	26.3 (7.26)	3.298	0.001	2.277
^∗∗^Emotional response	7.4 (1.55)	16.5 (6.51)	3.352	0.001	1.923
^∗∗^Emotional awareness	6.2 (1.64)	9.4 (2.35)	3.288	0.001	0.592
^∗∗^Overall score	51.9 (8.85)	78.4 (13.01)	3.235	0.001	2.382

^∗^ *p* < 0.05, ^∗∗^*p* < 0.01; BRIEF-2: Behavior Rating Inventory of Executive Function-2; CQIA-2: Caregiver Questionnaire for Interoceptive Awareness-2.

## Data Availability

Data supporting the results of this study is not available to protect participant privacy.

## Supplementary Materials

Supplementary Materials TREND statement checklist.
